# Why Do You Trust News? The Event-Related Potential Evidence of Media Channel and News Type

**DOI:** 10.3389/fpsyg.2021.663485

**Published:** 2021-04-14

**Authors:** Bonai Fan, Sifang Liu, Guanxiong Pei, Yufei Wu, Lian Zhu

**Affiliations:** ^1^Zhejiang Research Base for China's Non-Public Economic Personages, Ningbo University, Ningbo, China; ^2^Institute for Public Policy of Zhejiang University, Hangzhou, China; ^3^State Development & Investment Corp., Ltd. (SDIC) Beijing, China; ^4^Zhejiang Lab, Research Center for Advanced AI Theory, Hangzhou, China; ^5^School of Journalism and Communication, Shanghai International Studies University, Shanghai, China

**Keywords:** trust in news, media channel, news type, event-related potential, N100, P200

## Abstract

Media is the principal source of public information, and people's trust in news has been a critical mechanism in social cohesion. In recent years, the vast growth of new media (e.g., internet news portals) has brought huge change to the way information is conveyed, cannibalizing much of the space of traditional media (e.g., traditional newspapers). This has led to renewed attention on media credibility. The study aims to explore the impact of media channel on trust in news and examine the role of news type. Twenty-six participants were asked to make trust–distrust decisions after reading a variety of news headlines from different media channels while undergoing electroencephalography (EEG) monitoring. The electrophysiological results showed that, for hard news (e.g., important news related to public life), the new media condition elicited smaller N100 and larger P200 amplitudes than the traditional media condition. However, for soft news (e.g., entertainment, and non-related to vital interest), there was no significant difference. The study suggests that the fitness of media channel and news type may influence the evaluation of news, impacting participants' affective arousal and attention allocation in the early stage and influencing trust in news. These results provide neurocognitive evidence of individuals' trust toward hard and soft news consumed via different media channels, yielding new insights into trust in media and contributing to media trust theory.

## Introduction

Since media is the main source of everyday public information, people's trust in news has been a critical mechanism in social cohesion and key concern in the mass communication era (Hanitzsch et al., 2018). In communication studies, trust in media has been conceptualized as the extent to which the media is believed by news consumers, with the principal focus being the channel through which content is delivered (Finn and Gil de Zúñiga, 2011). The trustworthiness of media channels has engaged scholars' interest over the past few decades. The first comprehensive analysis of media credibility demonstrated that television news was typically regarded as more truthful than print news (Westley and Severin, 1964). Subsequent comparative studies of people's trust in news mainly focused on the credibility competition between newspapers and broadcast (Gaziano and McGrath, 1986; Newhagen and Nass, 1989). In recent years, with the rapid development of new media, the way in which information is conveyed and consumed has undergone dramatic changes. Comparative studies of the relative credibility of traditional media (e.g., traditional newspapers) and new media (e.g., internet news portals) have produced a number of interesting findings (Apejoye, 2015; Arrese and Kaufmann-Argueta, 2016; Kerunga et al., 2020). Studies found that people rated traditional media as the most trustworthy (e.g., Flanagin and Metzger, 2000). Bolstering this view, some people rated new media as less accurate but unbiased compared with information from traditional media due to the lack of effective constraints of new media (e.g., Salaudeen and Onyechi, 2020).

Apart from the different media channels, there are also two types of news, hard news and soft news. Hard news typically relates to international affairs, politics, business, economics, and finance (Reinemann et al., 2012; Newman et al., 2016). It is about what is at stake for the nation and the whole world and of vital importance for public daily life (Pearson and Knobloch-Westerwick, 2018). By contrast, soft news deals with celebrities, entertainment, arts, scandals, and sports (Reinemann et al., 2012; Newman et al., 2016). It relates to people's personal lives and can be trivial, emotionally evocative, and sensational (Reinemann et al., 2012; Kalogeropoulos et al., 2017; Otto et al., 2017). It was reported that hard news is considered more suitable for publication in traditional media, while soft news is more acceptable in new media (Fletcher and Nielsen, 2019). This led us to propose that people may have different levels of trust in different types of news (i.e., hard news and soft news) when the news headlines are presented in different media channels (i.e., traditional media and new media). However, there is still no direct evidence that the fitness between media channel and news type will influence the trust perception. Therefore, to address this gap, this study focuses on whether and how the news type/news channel fits impacts trust in news.

Concerning the measurements for self-report, researchers have failed to agree on the main dimensions of the concept, and no standardized scale can be directly used (Calvo-Porral et al., 2014; Scharkow and Bachl, 2017). What is more, people may not express their true opinion for several reasons, such as social desirability, cognitive bias, or political reasons (Plassmann et al., 2015). Thus, a more valid approach to measuring trust in news involves observing neural activity (Pan et al., 2018; Wan et al., 2018).

Functional magnetic resonance imaging (fMRI) and event-related potentials (ERPs) are the most widely used tools to identify the neural mechanisms underlying trust behavior. The advantage of ERPs is that they measure brain activity over a period of time and are therefore more suitable for assessing news consumption. For example, by using fMRI, Meyer-Lindenberg (2008) demonstrates that oxytocin and cortisol are closely correlated with trust. Studies have also shown that conditional trust selectively activates the ventral tegmental area, whereas unconditional trust selectively activates the septal area (Krueger et al., 2007). Compared with fMRI studies, ERPs provide a more real-time, temporally precise look. For instance, a coin-toss game, designed to study trust behavior and its neural mechanisms, provided evidence that both feedback-related negativity (FRN) and P300 can reflect the extent of trust (Long et al., 2012).

Measurements based on the registration of neuro-physiological parameters result in objective data, which can reveal what is happening in the brain and increase our understanding of the neural mechanisms underlying trust behavior, and the relevant emotional processes (Ma et al., 2015). Therefore, the current study intends to explore the interaction effect of media channels (new media vs. traditional media) and news types (soft news vs. hard news) on trust in news by using ERPs, which offer an opportunity to assess facets of cerebral functioning during news processing.

N100 is an early negative ERP component induced by emotional information, which reaches its peak at ~130–150 ms after the onset of an emotional stimulus at frontal-central sites (Yu et al., 2018). According to previous studies, N1 is considered to be an index of affective arousal (Lin et al., 2018). The amplitude of this component was larger for emotional than for neutral stimuli (Löfberg et al., 2014; Zinchenko et al., 2015). It was also shown that pleasant targets were preferentially processed in contrast to other targets shown by augmented N100 amplitudes (Garrido-Vásquez Schmidt et al., 2018). We hypothesized that a good fitness between media channel and news type would increase positive arousal and amplify N100.

P200 is a positive-going ERP component over frontal regions, which means a small voltage reflects a smaller P200 effect and can reflect the early assessment of stimuli (Garrido-Vásquez Schmidt et al., 2018). It typically peaks at ~250–350 ms after the onset of a stimulus (Jin et al., 2018). According to previous studies, P200 is an attention-related component, and larger P2 amplitudes will be elicited for the stimuli gaining more attention resources (Jin et al., 2017). Furthermore, it was reported that negative stimuli gained more attention resources than positive stimuli, which can result in larger P200 amplitudes (Wang et al., 2012). For example, Wang et al. (2012) found that less beautiful pendants elicited larger P200 amplitude than more beautiful ones. The author explained that the less beautiful pendants attracted more attention resources. Furthermore, Jin et al. (2017) also found negatively framed descriptions of product induced larger P200 amplitude than positively framed descriptions. They likewise supposed that this is because the negative framing attracted more attention. In the current study, we hypothesized that an inappropriate combination of media channel and news type would receive more attention allocation and induce larger P200 amplitude.

To sum up, we aim to explore the effect of media channel and news type on people's trust in news, by employing the prime-probe paradigm and ERPs. We supposed that the fitness of media channel and news type will influence trust in news, which would reflect in behavioral self-report trust score and deflection of N100 and P200 amplitude.

## Materials and Methods

### Participants

Twenty-six healthy undergraduates (14 female, mean age = 21.7, S.D. = 1.95) were recruited to participate in the experiment. All participants were self-reportedly right-handed and had normal or corrected-to-normal vision without prior history of neurological or psychiatric illness. All procedures performed in this experiment were in accordance with the ethical standards of the institutional research committee and with the 1964 Declaration of Helsinki and its later amendments or comparable ethical standards (World Medical Association, 2014). All participants provided written informed consent before the experiments began and received a payment of 40 yuan after the experiment. The current study was approved by the Internal Review Board.

### Stimuli and Material

A prime-probe paradigm was used in the experiment, which means that the prime stimuli and probe stimuli appeared in sequence within one trial procedure. Experimental materials comprised 160 stimuli, which consisted of the names of 16 media organizations (traditional newspaper vs. internet news portal) × 10 news headlines (hard news vs. soft news).

As for the selection of media names, we invited five experts to brainstorm and listed 20 representative names of media organizations for each media channel (i.e., traditional newspaper and internet news portal). Next, 50 students from different majors ranked the media sources according to their familiarity with the media names. The top eight from each media category were selected as the prime stimuli. Thus, the prime stimuli (S1) consisted of eight traditional newspaper names and eight internet news portal names in China. The definitions of the concepts “internet news portal channel” and “traditional newspaper channel” will change over time.

Moreover, the probe stimuli (S2) were five news headlines from each category (i.e., hard and soft). Because the subjects might have had prejudices about some news sources and preexisting views, it was necessary to select neutral news or to create soft news to avoid these potential influences. First, for the selection of hard news, we sought expert advice to ensure the validity of hard news features. Then, we chose hard news headlines from the State Statistics Bureau website (http://www.stats.gov.cn/), which were vetted through two rounds of selection. In the first round of selection, we asked five experts to choose 20 pieces of neutral economic information from the website and rewrite them into news headlines form with their domain knowledge and skills. Twenty news headlines went into the second round of selection in which 50 students from different majors scored their neutrality, and the top five most neutral news titles became the final stimuli. Second, for the selection of soft news, we created soft news to avoid prejudices about some news sources and preexisting views. The selection procedure also involved two rounds of selection. In the first round of selection, we asked five experts to create 20 pieces of soft news with their domain knowledge and skills. The selection method for the second round was the same as for hard news selection, resulting in the top five most neutral soft news titles becoming the final stimuli.

**Figure 1:**
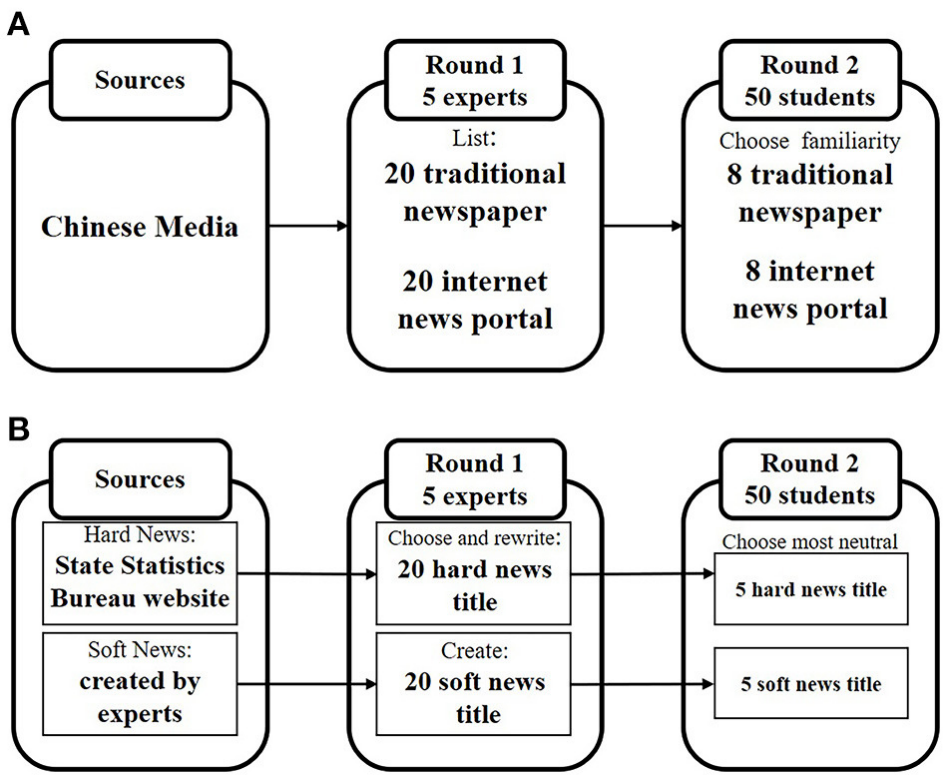
Selection procedure of experiment stimulus materials. **(A)** media name; **(B)** news title.

We now had four conditions: internet news portal-hard news, internet news portal-soft news, traditional newspaper-hard news, and traditional newspaper-soft news. The selection procedure for the media channel is shown in Figure 1A, while the selection procedure for the news headlines is shown in Figure 1B.

All media names and news titles were displayed in image form (bmp) in a SimSun 12-point font. The media names were no more than five Chinese characters and no more than 15 Chinese characters for news headlines. Each stimulus picture with a light gray background and a media name or news title in the center was digitized at 360 × 270 pixels.

### Experimental Procedure

The participants were comfortably seated in a dimly lit, sound-attenuated, and electrically shielded room. The stimuli were designed and presented using the E-Prime 2.0 software package (Psychology Software Tools, Pittsburgh, PA, USA). Each stimulus was displayed at the center of a screen that was placed 100 cm from the subjects. Stimuli were presented in the center of a computer screen with a visual angle of 2.58° × 2.4°.

**Figure 2:**
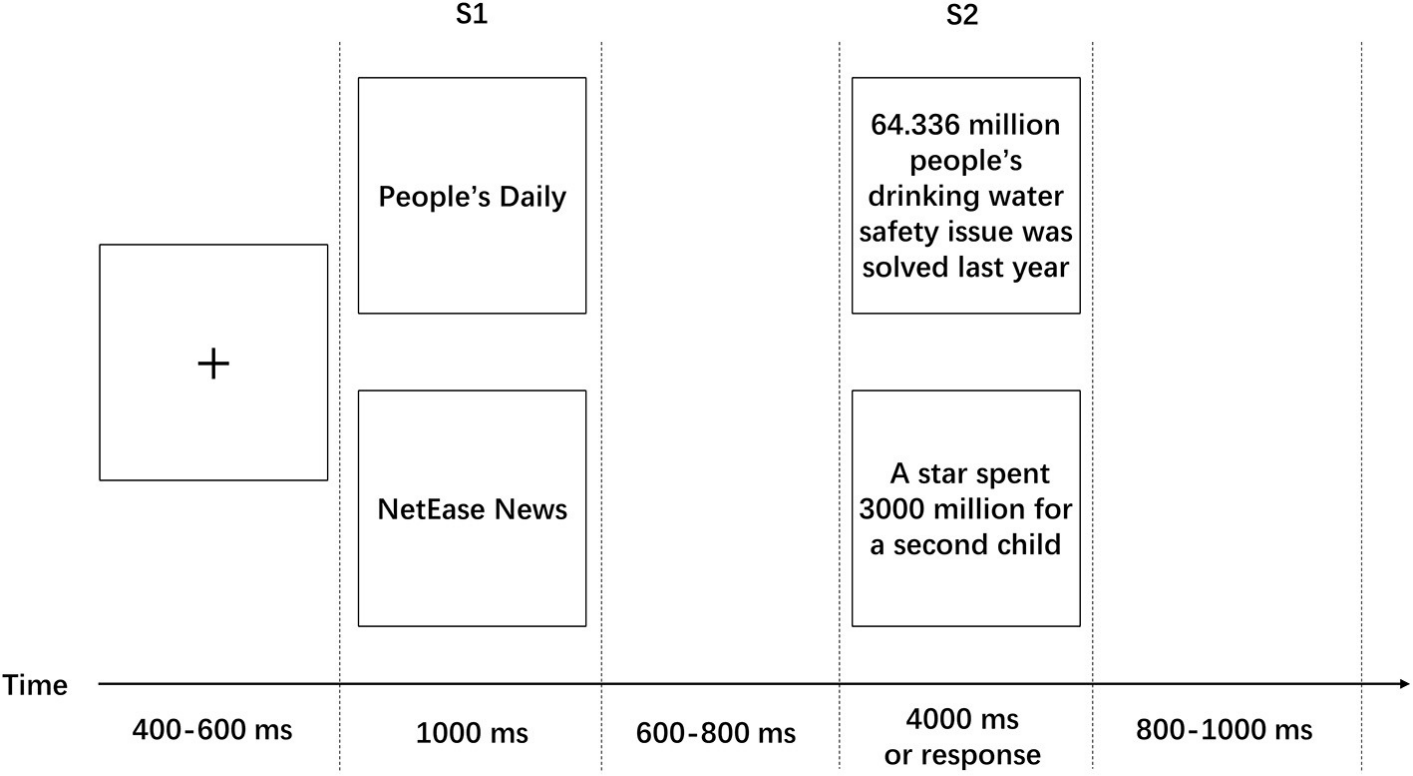
Single trial of the experimental procedure: Participants first observed either a new media channel or a traditional media channel before the presentation of a hard news or soft news item. They were asked to press a button to indicate whether or not they would trust the news presented in 4,000 ms. Participants' electroencephalograms (EEGs) were recorded throughout the experiment.

Prior to the experiment, the participants were instructed to study the experimental task and the procedure, and they subsequently performed eight practice trials to become familiar with the experiment. The procedure of a single trial is illustrated in Figure 2. Each trial was initiated by a fixation point that was presented for a period that was randomly varied from 400 to 600 ms (average = 500 ms). According to previous studies with similar settings (Ma et al., 2010), the average allocated time for a single Chinese character is about 200 ms; thus, we set the allocated times of S1 and S2 based on this. The name of one media channel (S1) was displayed for 1,000 ms after fixation. A blank screen was then presented between the prime and probe stimuli and lasted for a random duration of between 600 and 800 ms. Next, the S2 (news title) was presented for the longest duration of 4,000 ms and disappeared immediately when the subjects pressed a key after making their decision. Finally, an ISI (interstimulus interval, which means the interval between offset of the previous stimulus and the onset of the next stimulus) with a duration of 800–1,000 ms (varied randomly) was presented as the link between two trials. The task of the participants was to report their decisions regarding whether they would trust the news presented as S2 if that news was reported by the media source presented in S1. The key assignment was counterbalanced on the group level and assigned randomly across subjects. Half of the participants pressed the “1” key to indicate “trust” and the “3” key to indicate “distrust,” and the other half did the opposite.

### Electroencephalography Data Recording

A cap with 64 Ag/AgCl electrodes and a Neuroscan Synamp2 Amplifier (Scan 4.3.1; Neurosoft Labs Inc.) recorded the electroencephalography (EEG) signals. The experiment used a cephalic (forehead) location (between FPz and Fz) as the ground and the left mastoid for reference according to the standard international 10–20 system. Electrooculograms (EOGs) were recorded from electrodes placed to record the horizontal EOGs of both eyes (10 mm from the lateral canthi) and the vertical EOGs (above and below the left eye). The experiment was only initiated when the electrode impedances were maintained below 5 kΩ.

The data were transferred off-line and averaged for the left and right mastoid references. EOG artifacts were corrected off-line for all participants, using the method proposed by Semlitsch et al. (1986). A low-pass filter at 30 Hz and a high-pass filter at 0.1 Hz (24 dB/octave) were used to digitally filter the averaged ERPs. The EEG recordings were segmented for the epoch from 200 ms before the onset of the target appearing on the video monitor to 800 ms after this onset, and the first pretarget 200 ms was used as a baseline. Trials were excluded in the following circumstances: amplifier clipping, bursts of electromyography activity, and peak-to-peak deflections exceeding ±100 μV. Every participant's EEG recordings over each recording site were separately averaged within their experimental conditions, namely, internet news portal and hard news (NH), internet news portal and soft news (NS), traditional newspaper and hard news (TH), and traditional newspaper and soft news (TS).

### Data Analysis

Reaction time (RT) was analyzed using a 2 media channel (traditional media/new media) × 2 news category (hard news/soft news) ANOVA. The same 2 media channel (traditional newspaper/internet news portal) × 2 news category (hard news/soft news) ANOVA was conducted to measure the trust rate (TR). For brain activity analysis, we chose the time windows of the two ERP components on the basis of the visual observations of the grand average waveforms and the guidelines provided by the Picton team (Picton et al., 2000). According to Picton et al. (2000), we determine the peak of the grand average waveforms based on visual observation, and then we select a certain time around the peak to determine the time window of this component. The time windows of N100 and P200 were 160–210 and 210–270 ms after onset, respectively. The nine electrodes were collected because it has been proven and is widely accepted that both N100 (Yu et al., 2018) and P200 (Wang et al., 2012) can usually be found in the frontal-central area. Then, two media channels (traditional newspaper/internet news portal) × 2 news categories (hard news/soft news) × 9 electrodes (F1/z/2, FC1/z/2, C1/z/2) ANOVAs were performed for the N100 and P200. Simple-effect analyses were used to address the interactive effects. The Greenhouse–Geisser (Greenhouse and Geisser, 1959) correction was applied for the violation of the assumption of sphericity in the appropriate parts of the ANOVA (uncorrected degrees of freedom are reported with the ε and the corrected *p*-values). A simple-effect analysis was applied if the interactive effect was significant. We used partial eta-squared (η^2^_*p*_) values to demonstrate the effect size in ANOVA models, where.05 represents a small effect,.1 represents a medium effect, and 2 represents a large effect (Cohen, 1988; Muller and Barton, 1989).

## Results

### Behavioral Results

The RT and TR were the two indicators used to observe participants' behaviors. One piece of behavioral data was missing. We conducted a 2 media channel (traditional newspaper/internet news portal) × 2 news type (hard news/soft news) ANOVA to analyze whether the media channel and news type individually or interactively exhibited any significant effect on the RTs and TRs.

**Figure 3:**
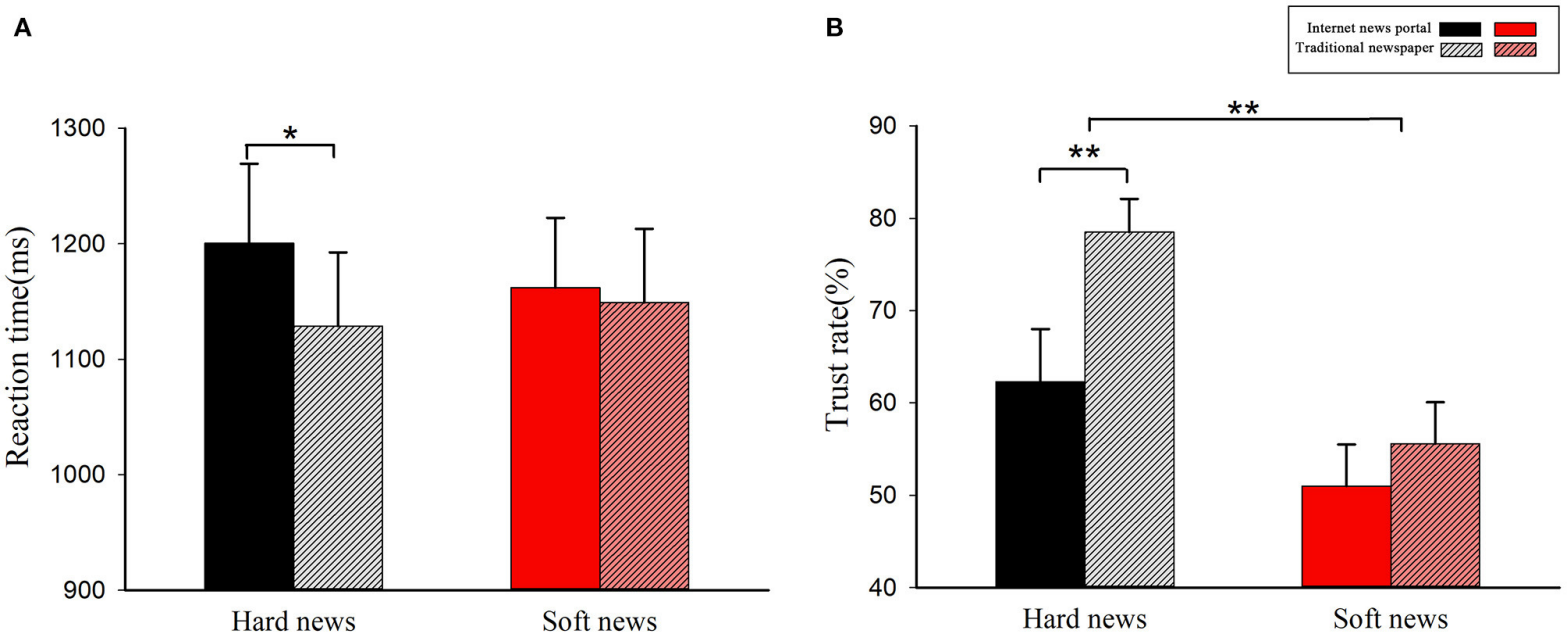
Behavioral results of the reaction time (RT) and ratio of “trust” answer (TR): **(A)** the reaction time of the two media channels with two news items (NH vs. NS vs. TH vs. TS); **(B)** the ratio of “trust” answer to two media channels with two news items (NH vs. NS vs. TH vs. TS). **p < 0.01; *p < 0.05.

The results showed that the main effect of news type [*F*_(1, 25)_ = 0.03, *p* > 0.1, η^2^_*p*_ < 0.01] and media type [*F*_(1, 25)_ = 2.79, *p* > 0.05, η^2^_*p*_ = 0.10] had no significant impact on RT. What is more, a significant interacted effect [*F*_(1, 25)_ = 5.66, *p* < 0.01, η^2^_*p*_ = 0.19] for the media channel and news type was observed. Simple-effect analyses revealed that, when dealing with hard news, participants responded significantly slower [*F*_(1, 25)_ = 6.08, *p* < 0.05, η^2^_*p*_ = 0.20] to internet news portals [NH: mean = 1,180.51 ms, S.E. = 69.15] than to traditional newspapers [TH: mean = 1,117.47 ms, S.E. = 62.43]. However, the effect of the media channel was not significant when dealing with soft news [*F*_(1, 24)_ = 0.16, *p* > 0.1, η^2^_*p*_ < 0.01]. The result is shown in Figure 3A.

The same analysis was conducted to measure TR. The results showed that the main effect of media type [*F*_(1, 25)_ = 3.56, *p* > 0.05, η^2^_*p*_ = 0.13] was not significant. However, the news type had significant main effects [*F*_(1, 25)_ = 12.21, *p* < 0.01, η^2^_*p*_ = 0.33], which means participants considered hard news (H: mean = 68.1%, S.E. = 4.4%) more credible than soft news (S: mean = 52.8%, S.E. = 3.0%). What is more, a significant interacted effect [*F*_(1, 25)_ = 4.29, *p* < 0.05, η^2^_*p*_ = 0.33] for the media channel and news type was observed.

A simple-effect analysis was performed. The results revealed that under the hard news condition [*F*_(1, 25)_ = 8.33, *p* < 0.01, η^2^_*p*_ = 0.25], participants considered traditional newspapers (H: mean = 75.6%, S.E. = 4.5%) more credible than internet news portals (S: mean = 60.7%, S.E. = 5.7%). However, the effect of the media channel was not significant [*F*_(1, 25)_ = 0.52, *p* > 0.1, η^2^_*p*_ = 0.2] with soft news. The result is shown in Figure 3B. Furthermore, when the media channel was fixed with respect to traditional newspapers, the news type was significant [*F*_(1, 25)_ = 17.26, *p* < 0.001, η^2^_*p*_ = 0.41], which means participants considered hard news (H: mean = 75.6%, S.E. = 4.5%) more credible than soft news (S: mean = 55.0%, S.E. = 4.4%). However, the effect of the news type was not significant [*F*_(1, 25)_ = 3.84, *p* > 0.05, η^2^_*p*_ = 0.13] with internet news portals.

### Event-Related Potential Results

#### N100

The results of a three-way 2 (traditional newspaper vs. internet news portal) × 2 (news type: hard vs. soft) × 9 (electrode: F1/z/2, FC1/z/2, C1/z/2) ANOVA of the N100 in the time window from 160 to 210 ms exhibited a significant main effect for news type [*F*_(1, 25)_ = 4.63, *p* < 0.05, η^2^_*p*_ = 0.16] and an interaction effect of media channel and news type [*F*_(1, 25)_ = 6.49, *p* < 0.05, η^2^_*p*_ = 0.21], whereas the main effect for the media channel was not significant [*F*_(1, 25**)**_ = 0.72, *p* > 0.1, η^2^_*p*_ = 0.03], as shown in Figure 3. The main effect for news type meant that soft news (mean = −0.23 μV, S.E. = 0.09) elicited larger negative amplitude than hard news (mean = −0.13 μV, S.E. = 0.10).

Therefore, a simple-effect analysis was performed to determine whether the media channel and news type had a significant interaction effect on each other. When the media channel was fixed with respect to internet news portal, the news type was significant [*F*_(1, 25)_ = 11.10, *p* < 0.01, η^2^_*p*_ = 0.31]. The mean amplitude of the N100 in response to soft news (NS: mean = −0.26 μV, S.E. = 0.10) was more negative than in response to hard news (NH: mean = −0.07 μV, S.E. = 0.10). However, the effect of news category was not significant [*F*_(1, 25)_ = 0.08, *p* > 0.1 η^2^_*p*_ < 0.01] with traditional newspapers.

Another interesting finding was that the media channel had a significant effect on the N100 amplitude when the news type was fixed to be hard news [*F*_(1, 25)_ = 4.68, *p* < 0.05, η^2^_*p*_ = 0.16]. Moreover, the mean amplitude of the N100 in response to traditional newspapers (TH: mean = −0.19 μV, S.E. = 0.11) was more negative than in response to internet news portals (NH: mean = −0.07 μV, S.E. = 0.10). What is more, the effect of the media channel category was not significant [*F*_(1, 25)_ = 1.67, *p* > 0.1, η^2^_*p*_ = 0.06] with soft news.

#### P200

**Figure 4:**
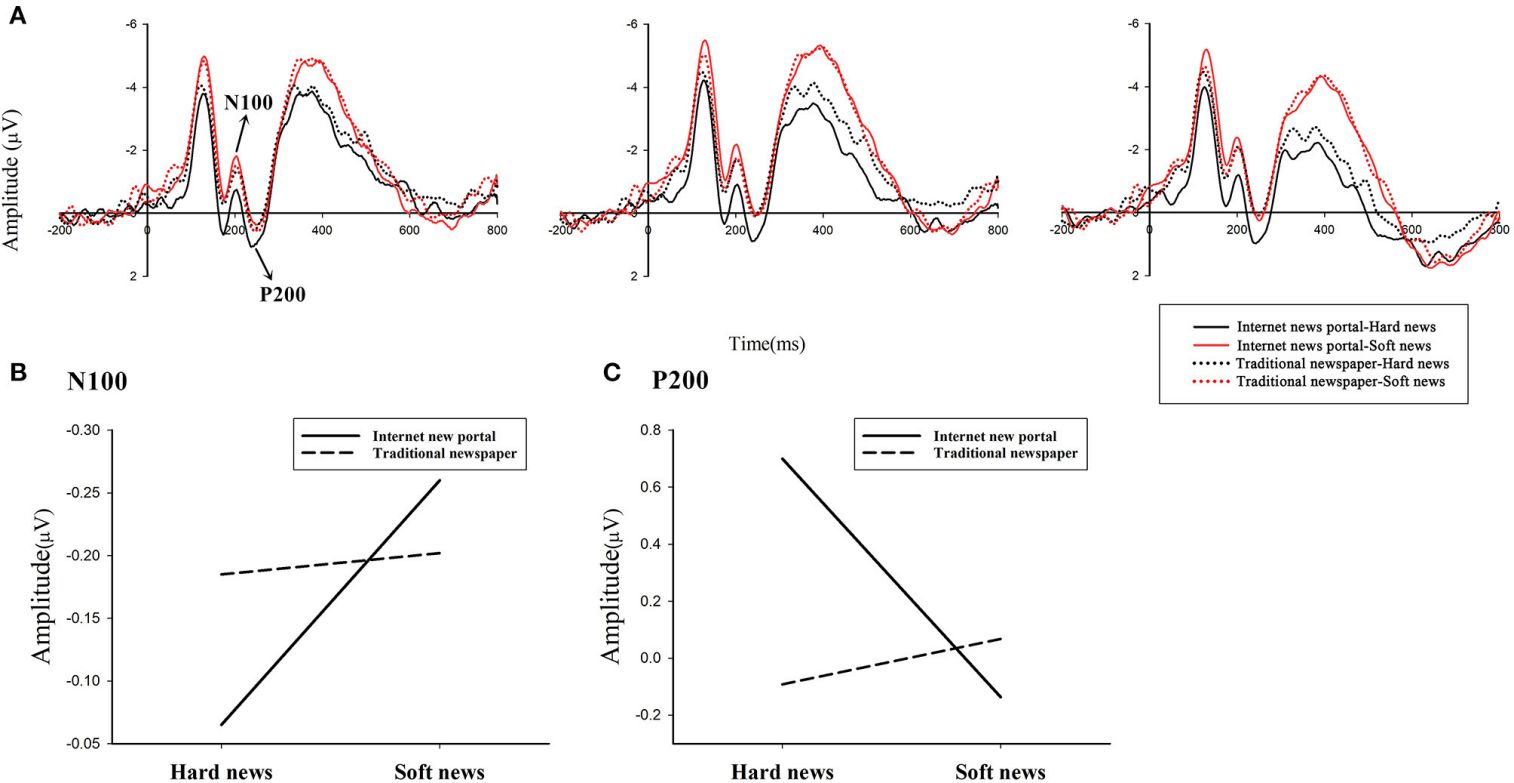
Grand-average N100 and P200 ERP waveforms elicited by news headlines in the three electrodes (Fz, FCz, and Cz): **(A)** the comparison of different media channels presenting different types of news. **(B)** Interaction effect between media channel and news type on N100. **(C)** Interaction effect between media channel and news type on P200.

The results of a three-way 2 × 2 × 9 ANOVA of the P200 exhibited no significant main effect for either media channel [*F*_(1, 25)_ = 1.37, *p* > 0.1, η^2^_*p*_ = 0.05] or news type [*F*_(1, 25)_ = 1.35, *p* > 0.1, η^2^_*p*_ = 0.05], whereas the interaction effect of media channel and news type was significant [*F*_(1, 25)_ = 5.51, *p* < 0.05, η^2^_*p*_ = 0.15], as shown in Figure 4.

Therefore, a simple-effect analysis was performed. When the media channel was fixed with respect to traditional newspapers, the traditional type was not significant [*F*_(1, 25)_ = 0.22, *p* > 0.1, η^2^_*p*_ < 0.01]. However, the effect of news types was significant [*F*_(1, 25)_ = 4.23, *p* < 0.05, η^2^_*p*_ = 0.15] with internet news portals. The mean amplitude of the P200 in response to hard news (NH: mean = 0.70 μV, *S.E*. = 0.74) was larger than in response to soft news (NS: mean = −0.14 μV, S.E. = 0.74).

Another interesting finding was that the media channel had a significant effect on the P200 amplitude when the news type was fixed to be hard news [*F*_(1, 25)_ = 6.01, *p* < 0.05, η^2^_*p*_ = 0.19]. Moreover, the mean amplitude of the P200 in response to internet news portals (NH: mean = 0.70 μV, *S.E*. = 0.74) was larger than in response to traditional newspapers (TH: mean = −0.09 μV, S.E. = 0.81). However, the effect of the media channel category was not significant [*F*_(1, 25)_ = 0.31, *p* > 0.1, η^2^_*p*_ = 0.01] with soft news.

### Discussion

The purpose of this study was to clarify the influence of media channel (traditional media: traditional newspaper vs. new media: internet news portal) and news type (hard news vs. soft news) on trust in news. Our behavioral results showed that hard news was rated as being more credible in traditional media than in new media but that there was no significant difference for soft news. The results of the current study are consistent with previous studies, which supposed that news from traditional media was considered to be more credible than news from new media, especially for the hard news. It was reported that the credibility of online news sources and the reliability of their information are widely questioned and that people often search for mass media with parallel content to ensure the truth of news they have just learned (Medhi, 2019). This is because traditional media strictly follows an inherent standard and offers more serious, exhaustive, and in-depth information, which is more credible and trustworthy. By contrast, people remain skeptical of the internet as a hard news resource, because of the lack of gate-keeping and fact-checking in online information publishing (Metzger and Flanagin, 2015). Thus, participants may hesitate with their trust ratings, especially when hard news was paired with new media.

What is more, we also found that hard news is more credible than soft news, especially in the traditional media. Hard news usually has a high level of newsworthiness because of its close association with politics, economics, and social matters (Reinemann et al., 2012; Newman et al., 2016). It is supposed to be strictly objective, and the journalist is not supposed to provide their views on the story. Put it another way, hard news is usually reported in a thematic and impersonal manner and is not sensational in its style. However, soft news refers to news with a low level of substantive informational value, e.g., gossip, human interest stories, and offbeat events, and is subjective, personality-centered, and always reported in an episodic manner (Reinemann et al., 2012). Thus, compared with soft news, hard news is more factual and may be regarded as more credible. In line with our findings, a previous study also showed that TV viewers recognize hard news stories as being more credible than soft news stories (Miller and Kurpius, 2010).

As far as the brain is concerned, we applied ERP to recognize neural components associated with trustworthiness decision-making processes and found that the influence of media channel and news type on trust in news could be reflected by the early frontal N100 and P200, a hypothesis supported by our behavioral results. In our study, N100 was found to be larger in the soft news than in hard news when reported in new media. As we stated in the *Introduction*, N100 is believed to be compulsory and driven by sensory-related procedures (Lin et al., 2018). Emotions can be recognized at a very early stage of processing as indexed by the frontal N100 component (Garrido-Vásquez Schmidt et al., 2018). It was also argued that the amplitude of N100 was influenced by arousal level (Lin et al., 2018). It was larger for emotional than for neutral stimuli (Löfberg et al., 2014; Zinchenko et al., 2015). Therefore, in the current study, the enhanced frontal negative activation may be induced by an emotionally salient combination of new media and soft news. It indicates higher positive emotional arousal and demonstrates that new media is more suitable to the publication of soft news. What is more, N100 amplitude was more negative in traditional media than in new media when reporting hard news, which may illustrate that hard news is more appropriate and acceptable in traditional media. People still tend to trust traditional media more in terms of its combination with hard news, for both of which participants had high credibility expectations.

Moreover, the P200 component results indicate that for hard news, new media elicited larger P200 amplitude than traditional media. As mentioned in the *Introduction*, frontal P200 is regarded as the index of the attention-related process. Enlarged P200 amplitudes indicate attentional enhancement or the involvement of more attention resources (Wang et al., 2012). Therefore, the current results may reveal that hard news reported in new media will attract more attention than that reported in traditional media. This may be because the match between channel and news is generally accepted as the essence of trust (Tsfati and Cohen, 2012). We make the inference that hard news is much more suited to traditional media than new media; hence, hard news reported in new media would bring mismatched perception and arousal attention, which reflect in larger P200. However, for soft news, there is no difference between traditional media and new media, which revealed that there is no attentional difference for soft news in different media channels. This is consistent with previous studies that suggested that compared with new media, traditional media has greater credibility (Idid et al., 2019; Salaudeen and Onyechi, 2020).

Based on this examination of the overall neural processes involved in trust and distrust decisions about news, we can divide this process into two phases. The first phase is the ~100 ms period during which the subjects saw the stimulus, reflected in the amplitude of N100 in the early automatic stage. The second phase is the ~200 ms period after the first glance at the news headline and the period of deeper thinking about the content, reflected in the amplitude of P200. According to the N100 and P200 results, it was obvious that, in the first phase, the fitness of media channel and news type influenced the level of affective arousal. In the second stage, the fitness influenced the degree of attention allocation. This N100–P200 complex demonstrates the underlying neural mechanism involved in the evaluation and decision-making process regarding trust in news, highlighting the importance of an appropriate combination of media channel and news type.

One limitation is that our results are based on a sample of students assumed to be familiar with all kinds of media channel. Previous studies demonstrated that the amount of time spent on the internet was a strong predictor of the credibility rating of new media. There is a positive association between internet use and online news credibility (Salaudeen and Onyechi, 2020). Future studies could be conducted among the younger generation in China who are not regular and faithful users of traditional media. Another limitation is that we only looked at traditional newspapers as representative of traditional media and internet news portals as representative of new media. In the future, more media channels (e.g., broadcast and news apps) will be considered.

## Conclusions

In summary, the current study explored the interaction effect of media channel and news type on trust in news. Behavioral results showed that participants considered hard news to be less credible when reported in new media than in traditional media. Further electrophysiological results showed that hard news under the new media condition induced smaller N100 and larger P200 amplitude than hard news under the traditional media condition. However, for soft news, there was no significant difference. Our results offer neurocognitive evidence of people's varied levels of trust toward news from different media channels, underlining the importance of an appropriate condition of media channel and news type.

## Data Availability Statement

The raw data supporting the conclusions of this article will be made available by the authors, without undue reservation.

## Ethics Statement

The studies involving human participants were reviewed and approved by Academy of Neuroeconomics and Neuromanagement, Ningbo University. The patients/participants provided their written informed consent to participate in this study.

## Author Contributions

BF made substantial contribution to the conceptualization, methodology, and writing-original draft preparation. LZ made substantial contribution to the conceptualization, methodology, data curation, and writing-revised draft preparation. SL made substantial contribution to data acquisition, analysis, and interpretation of data. GP and YW made substantial contribution to the analysis and interpretation of data. All authors contributed to the article and approved the submitted version.

## Conflict of Interest

SL was employed by the company SDIC Mining Investment Co., Ltd., Beijing, China. The remaining authors declare that the research was conducted in the absence of any commercial or financial relationships that could be construed as a potential conflict of interest.
